# Molecular Simulations of Disulfide-Rich Venom Peptides with Ion Channels and Membranes

**DOI:** 10.3390/molecules22030362

**Published:** 2017-02-27

**Authors:** Evelyne Deplazes

**Affiliations:** 1 School of Biomedical Sciences, Curtin Health Innovation Research Institute, Curtin University, Perth, WA 6102, Australia; evelyne.deplazes@curtin.edu.au; 2 School of Chemistry and Molecular Biosciences, The University of Queensland, Brisbane, QLD 4072, Australia

**Keywords:** venom peptides, ion channels, structure-based drug design, molecular dynamics simulations, molecular docking, molecular modelling, free energy calculations

## Abstract

Disulfide-rich peptides isolated from the venom of arthropods and marine animals are a rich source of potent and selective modulators of ion channels. This makes these peptides valuable lead molecules for the development of new drugs to treat neurological disorders. Consequently, much effort goes into understanding their mechanism of action. This paper presents an overview of how molecular simulations have been used to study the interactions of disulfide-rich venom peptides with ion channels and membranes. The review is focused on the use of docking, molecular dynamics simulations, and free energy calculations to (i) predict the structure of peptide-channel complexes; (ii) calculate binding free energies including the effect of peptide modifications; and (iii) study the membrane-binding properties of disulfide-rich venom peptides. The review concludes with a summary and outlook.

## 1. Introduction

**Figure 1:**
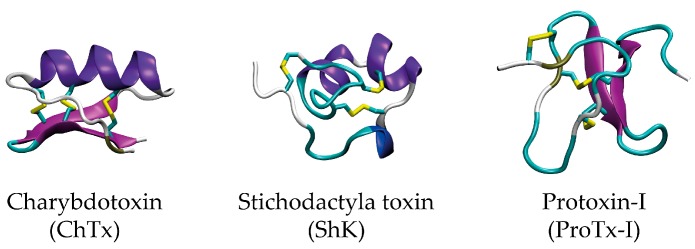
Structures of the disulfide-rich venom peptides Charybotoxin (ChTx, PDB-id 2CRD), Stichodactyla toxin (ShK, PDB-id 1ROO), and Protoxin-I (ProTx-I, PDB-id 2M9L). The well-defined secondary structure is stabilised by one or more disulfide bonds (yellow).

The venom of arthropods (e.g., spiders, scorpions, and centipedes) and marine animals (e.g., cone snails, jellyfishes, and sea anemones) are a rich source of biologically active molecules. A major component of these venoms are disulfide-rich peptides, also referred to as toxins. They are typically 10–60 amino acids long and fold into a well-defined secondary structure that is stabilised by multiple highly conserved disulfide bonds [1]. The peptides are highly resistant to extremes of solvents, pH, and temperature as well as to degradation by proteases. Figure 1 depicts the structure of three examples of such disulfide-rich peptides: Charybdotoxin (ChTx) isolated from the venom of the scorpion *Leiurus quinquestriatus hebraeus*, Stichodactyla toxin (ShK) from the Caribbean sea anemone *Stichodactyla helianthus*, and Protoxin-I (ProTx-I) from the Peruvian green-velvet tarantula *Thrixopelma pruriens.*

Many disulfide-rich venom peptides are potent and selective inhibitors of ion channels and other membrane-bound receptors. Among the most common molecular targets are voltage-gated Na^+^ (Na_V_) and K^+^ (K_V_) channels, voltage-sensitive Ca^2+^ channels, mechanosensitive channels, nicotinic acetylcholine receptors, transient receptor potential (TRP) cation channels, and acid sensing ion channels (ASICs) [2,3,4,5,6,7]. These channels and receptors are involved in almost all aspects of mammalian physiology and many of them are associated with pathophysiological conditions including autoimmune disorders, cardiac diseases, as well as neurological and musculoskeletal disorders. Disulfide-rich venom peptides are thus valuable pharmacological tools to examine the role of these ion channels and receptors in both physiological and pathological conditions [3,8] as well as lead molecules to develop new pharmaceuticals [2,4,6,7,9,10].

**Figure 2:**
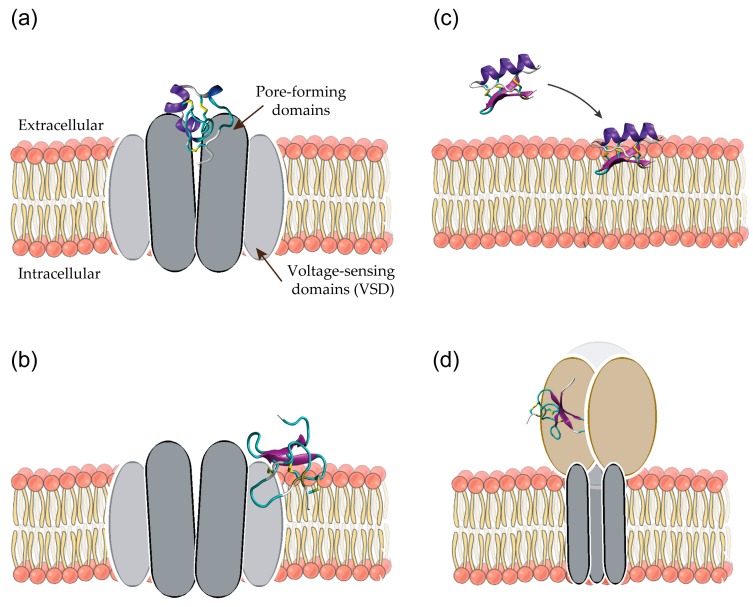
Schematic representation of binding sites and mechanisms of action for disulfide-rich venom peptides acting on voltage-gated ion channels (**a**,**b**) and acid sensing ion channels (ASICs) (**d**). (**a**) Pore blockers prevent ion conduction by binding to the pore-forming domains; (**b**) Gating modifiers alter the gating behaviour by binding to the voltage-sensing domains; (**c**) The mechanism of some gating modifiers involves the binding of the peptide to the cell membrane; (**d**) Most disulfide-rich venom peptides that inhibit ASICs bind to the large extracellular domain.

Translating venom peptides into therapeutically useful molecules often involves the use of structure-based and rational drug design approaches. For this, a detailed understanding of the structural, chemical, and biophysical properties of the peptide as well as its interactions with the target protein is required. While the specific interactions between a peptide and a given protein are unique, many peptides act via similar mechanisms as illustrated in Figure 2. Peptides that modulate voltage-gated ion channels (e.g., Na_V_ and K_V_) can act as pore blockers or gating modifiers. Pore blockers bind to the solvent-accessible pore domain of the channel and prevent the flow of ions (Figure 2a). Gating modifiers bind to the membrane-embedded voltage-sensing domains (VSDs) and thus alter the kinetics and gating behaviour of the channel (Figure 2b). As the VSDs are partially embedded into the membrane, the activity of some gating modifiers is related to their ability to bind to or partition into the membrane (Figure 2c) [11,12]. Peptides that modulate ASICs [8] mostly act by binding to the large extracellular domain where putative proton-sensing residues are located (Figure 2d). 

Biomolecular simulations and related molecular modelling approaches have been extensively used to understand the interactions of venom peptides with ion channels and membranes [13,14]. Increases in computing resources, more efficient algorithms, and improvements in the force fields used to model biomolecular systems have led to a significant increase in the size and complexity of the systems and time-scales that can be simulated. As a result, simulations of biomolecular systems have become more realistic and enable the calculation of macroscopic and structural properties that allow a direct comparison to experimental data [15]. 

Most simulation studies of disulfide-rich venom peptides have focused on understanding the interactions of peptides bound to voltage-gated Na_V_ and K_V_ channels [14,16,17] as well as ASICs [18,19,20,21] (see also Figure 2). This partially reflects the abundance of peptides that modulate these ion channels and their therapeutic potential but also the availability of high-resolution crystal structures for these proteins. The main focus of most simulation studies is characterising the binding mode of the peptide. Specifically, the aim is to identify the binding site on the channel and predict the pairwise peptide-channel interactions. This is mostly achieved using docking and molecular dynamics (MD) simulations. In addition, free energy calculations are used to predict binding affinities. Such studies might also include predicting the effect of modifications in the peptide on the binding mode or affinity. In addition, a number of studies have used MD simulations to investigate the interactions of disulfide-rich venom peptides and membranes. 

For the interactions predicted by the simulations to be of use for rational drug design, atomistic-level details are required (or are at least highly desirable). Furthermore, simulations should mimic a physiological environment. Thus, a typical simulation system consists of the peptide-channel complex either in water or embedded in a membrane, or a peptide bound to a membrane. This results in large simulation systems thus limiting the feasible simulation times to hundreds of nanoseconds. Yet the large number of potential interactions at the binding interface and the possible conformational changes induced by binding, either in the peptide or the channel, requires sampling of a large conformational space. All of this makes simulations of peptide-channel or peptide-membrane systems challenging and resource-intensive. Many of the simulation techniques described in this review are not (yet) suitable for screening approaches typically used during early stages of drug discovery. They are more applicable to characterising a small number of peptides during later-stage lead optimisation or to study model systems for understanding the fundamental processes that govern the binding of peptides to ion channels and membranes. 

In the remainder of this paper, simulations of disulfide-rich venom peptides are reviewed based on the questions the studies aim to address. The first section focuses on how docking and MD simulations have been used to predict the structure of peptide-channel complexes. The second section reviews studies that aim to estimate the binding affinity of a peptide for a given ion channel using a range of free energy methods. The third, and last, section outlines studies that investigate the binding of disulfide-rich venom peptides to membranes. The review concludes with a summary and outlook.

The studies mentioned in this review use methods that are based on the molecular mechanics approach. The general principles of these simulation techniques are only briefly described in the relevant sections. For detailed information and the underlying theory, the reader is referred to the appropriate literature. Also, challenges that are common to modelling biomolecular systems [15] such as the choice of force field [22,23], the use of enhanced sampling methods [24,25], the problem of convergence in MD simulations [26,27], or the evaluation of scoring functions in docking [28,29] are not covered in this review. Finally, the reader should be aware that there are other computational methods used to study venom peptides that are not discussed here (e.g., QSAR, knowledge-based potential methods for peptide-channel docking, or Brownian dynamics simulations [30,31,32]).

## 2. Simulation Studies of Peptide-Channel Complexes

Given that lack of high-resolution structures of peptide-channel complexes, its prediction is usually the first step in simulations studies. More specifically, the aim is to identify the binding site on the channel and to predict the pairwise peptide-channel interactions that make up the complex interface formed upon binding. This is mostly achieved using docking or MD simulations (or a combination of both). Besides computing resources, the choice of method and simulation protocols often depend on the availability of experimental data or knowledge of the binding mode from structurally related peptides. Once one or more potential binding modes have been identified the peptide-channel interactions are analysed with the aim to rationalise the peptide’s experimentally observed potency and selectivity for a specific channel. In some cases, additional experiments or simulations are required to distinguish between alternative structural models. Note that the binding of peptides is usually reversible. Thus, unless explicitly stated, the term ‘binding’ usually implies that by studying the formation of the peptide-channel complex one obtains insight into both the binding and unbinding event. 

### 2.1. Predicting the Structure of Peptide-Channel Complexes without Experimental Data

Even if the approximate binding site on the channel is known, it is unlikely for a truly ‘blind’ docking approach to accurately and reliably predict the structure of the peptide-channel complex [19,20,33,34,35]. While there has been significant progress in docking algorithms, the docking of a flexible peptide to a flexible protein remains challenging due to the complexity of the binding interface and the large number of possible interactions [36,37,38,39,40,41]. 

One way to increase the accuracy is to exploit the fact that structurally related peptides show similar binding modes. For example, many peptides that inhibit voltage-gated ion channels via a pore blocking mechanism (Figure 2a) do so by inserting a charged residue into the selectivity filter of the channel. Chen et al. [42] used this knowledge to ‘dock’ the α-conotoxin PIIIA to the bacterial sodium channel Na_V_Ab using unbiased MD simulations. The peptide was positioned close to the selectivity filter of the channel and in two out of three simulations the peptide was seen to spontaneously bind to the channel. Nevertheless, the simulations produced two very different binding modes and in the absence of experimental data an expensive calculation of the binding affinity using umbrella sampling simulations was required to differentiate between them. 

An alternative approach is to use low-resolution methods to sample a large number of potential binding modes that are later refined using atomistic simulations. For example, Wee et al. [43] used multi-scale MD simulation to study the interactions of the spider-venom peptide VsTx1 and the voltage sensing domain (VSD) of the bacterial potassium channel K_V_AP. Five independent, 3-μs long coarse-grained MD simulations were used to model the spontaneous binding of the peptide. A set of potential peptide-VSD complexes was subjected to atomistic MD simulations to refine the predicted binding interface. While four out of five simulations showed a consistent binding mode the predicted interactions only partially agreed with available experimental data. These examples highlight the high level of uncertainty in predicting peptide-channel structures without a priori knowledge of the binding interface. Without subsequent experimental validation, such structural models remain speculative. 

### 2.2. Predicting the Structure of Peptide-Channel Complexes Guided by Experimental Data

A commonly used approach to improve the accuracy of the structures from docking and MD simulations is to make use of experimental information regarding the binding interface [18]. Potential sources of data include alanine scanning mutagenesis, NMR or cross-linking experiments, and bioinformatics predictions based on secondary structure and sequence conservation or even partial electron densities. Experimental data can also be used to eliminate false positives during post processing. For example, Chen & Chung [44] used this approach to predict the complex formed by the scorpion-venom peptide OSK1 and K_V_1.3. Rigid-body docking was used to produce 600 potential binding poses from which solutions consistent with data from NMR experiments of a structurally related peptide were selected. It is worth noting that the selected binding poses represented less than 3% of all possible solutions and were not among the top-scored ones. The same authors also applied this approach to study the interactions of the peptides Css4 and Cn2 to the VSD of Na_V_1.2 and Na_V_1.6 [42]. For each peptide-VSD complex, 300 potential binding modes were generated and only poses in which residues known to be involved in binding are close to the VSD were selected. For each complex, only one unique binding mode was identified but they were ranked very low by the docking program. A similar approach has been used in many other studies [44,45,46,47,48,49,50,51,52,53]. In most of them, the selected binding poses were further refined using unrestrained MD simulations and in some cases free energy calculations were used to differentiate between binding modes. 

An alternative approach is to use information on the binding interface as restraints to guide the docking. Examples of restraint-driven docking programs for predicting peptide-channel complexes include BiGGER [54], HADDOCK [55,56,57], and Rosetta [58]. A number of studies used this approach to predict the complexes formed by pore blocking peptides and Na_V_ or K_V_ channels [47,48,49,59]. Chen et al. [60] developed a ‘comparative docking protocol’ that involves both blind and restraint-driven docking to predict the selectivity profiles of structurally related pore blocking peptides that bind to K_V_1.1, K_V_1.2, and K_V_1.3. The authors noted that the predicted binding poses are ‘qualitative informative’ and can help to rationalise selectivity profiles, but the docking scores are not accurate enough to rank peptides by their relative binding affinities. Saez et al. [61] used restraint-driven docking to predict the structure formed by the spider-venom peptide PcTx1 and the acid sensing ion channel 1a (ASIC1a). The predicted binding mode was a good first approximation consistent with the experimental data known at the time but a subsequent co-crystal structure of the PcTx1-ASIC1a complex showed that the peptide adopted a different orientation. Later, Deplazes et al. [18] used the same PcTx1-ASIC1a complex as a model system to assess the ability of restraint-driven docking to reliably predict the structure of peptide-channel complexes. By comparing over 240,000 docked structures, the study examined the effect of different combinations of restraints and input structures on the statistical likelihood of a structure predicted by restraint-driven docking to be of sufficient accuracy for rational drug design. While increasing the number of restraints improved the likelihood of finding a structure within a given accuracy, other factors such as shape complementarity and the force field also contributed to the accuracy. In addition, the results revealed large variations depending on the precise combination of residues used as restraints. 

Information on the binding interface can also be used to carry out MD simulations with distance restraints [62,63,64,65,66,67,68]. The benefit of restraint-driven MD simulations over docking is that the time-dependent structural dynamics of the entire system is explicitly included in the simulation and, if needed, the protein can be embedded in a membrane environment. This, however, comes at a considerable computational cost compared to docking. Many of the studies using restrained MD simulation involved pore blocking peptides binding to the selectivity filter of K_V_ channels. In most cases, a single distance restraint based on the conserved binding mode from structurally related peptides was used [62,63,64,65,68]. Eriksson et al. [67] used a more intricate simulation protocol to study the interaction between the peptide AgTx2 and the voltage-activated *Shaker* K^+^ channel. First, a series of high-temperature MD simulations with different sets of distance restraints deduced from double mutant cycles were used to produce a large number of potential binding modes. From this, a small set of ‘plausible binding mode candidates’ was selected and further validated using unrestrained MD simulations. A similar approach was used by Choudhary et al. [66] in which the strengths of the distance restraint was adjusted depending on the change in binding energies observed in double mutant cycle experiments. In both studies, a small number of potential binding modes were identified from which specific mutation experiments were suggested for validation.

The majority of studies using restraint-driven docking and MD simulations described above have focused on pore blocking peptides binding to the selectivity filter of Na_V_ or K_V_. As mentioned before, the general pharmacophore of these peptides is well established and conserved across a range of structurally related peptides [69]. In addition, the selectivity filter shows high sequence identity between subtypes of the same channel so that the experimentally observed selectivity profile of peptides is often the result of a small number of peptide-channel interactions. This provides a large amount of information to remove false positives from a set of potential solutions as well as data for restraints. Many other venom peptides, including gating modifiers of Na_V_ or K_V_ and peptides inhibiting ASICs, have been studied less extensively and it is not known whether the binding modes between structurally-related peptides is conserved. There is also less data on which peptide-channel interactions govern subtype selectivity. Consequently, the level of uncertainty when modelling these peptide-channel complexes is higher.

### 2.3. Predicting Binding Free Energy for Peptide-Channel Complexes 

Besides predicting the structure and pairwise interactions of peptide-channel complexes, the ability to calculate the binding free energy is one of the most powerful applications of MD simulations. The binding free energy provides an estimate of a peptide’s binding affinity for a given channel. Free energy calculations can also provide insight into the pathway of binding, including any conformational changes in the peptide or the channel induced by complex formation [70]. From statistical mechanics, we know that the probability of finding a molecular system in one state (A) or another state (B) is determined by the difference in free energy between states A and B. For condensed phase systems, the relevant state for most biological processes, differences in free energy (ΔG), can be obtained from averages over ensembles of atomic configurations such as equilibrium MD simulations [71,72,73]. The accurate calculation of ΔG requires sufficient sampling of all microstates that contribute to the free energy difference. This is very challenging to achieve for large systems such peptide-channel complexes. Thus, despite increases in computing power and improvements in methodologies, the accurate and reliable calculation of binding free energy is still one of the most challenging and resource-intensive tasks in biomolecular simulations. A large number of methods and techniques for free energy calculations have been developed. For a comparison of these methods, the theory behind them, as well as practical aspects related to free energy calculations the reader is referred to the numerous reviews on the subject [70,71,72,73,74,75].

For venom peptides, free energy calculations are used to predict the binding free energy (ΔG_b_). That is, the free energy difference between the peptide in solution and the peptide bound to the channel. This can be calculated from the potential of mean force using umbrella sampling simulations [72,74,76,77,78,79,80,81,82] or Jarzynski’s equality [76,77,83,84,85]. In addition, free energy calculations are used to estimate the effect of chemical modifications in the peptide (e.g., mutations or cyclisation) on its binding affinity. This is often obtained from free energy perturbation (FEP) or end-point methods such as the molecular mechanics Poisson-Boltzmann or generalized born surface area method (MM/PBSA and MM/GBSA, respectively) [86,87,88,89,90].

#### 2.3.1. Predicting Binding Free Energies from the Potential of Mean Force 

**Figure 3:**
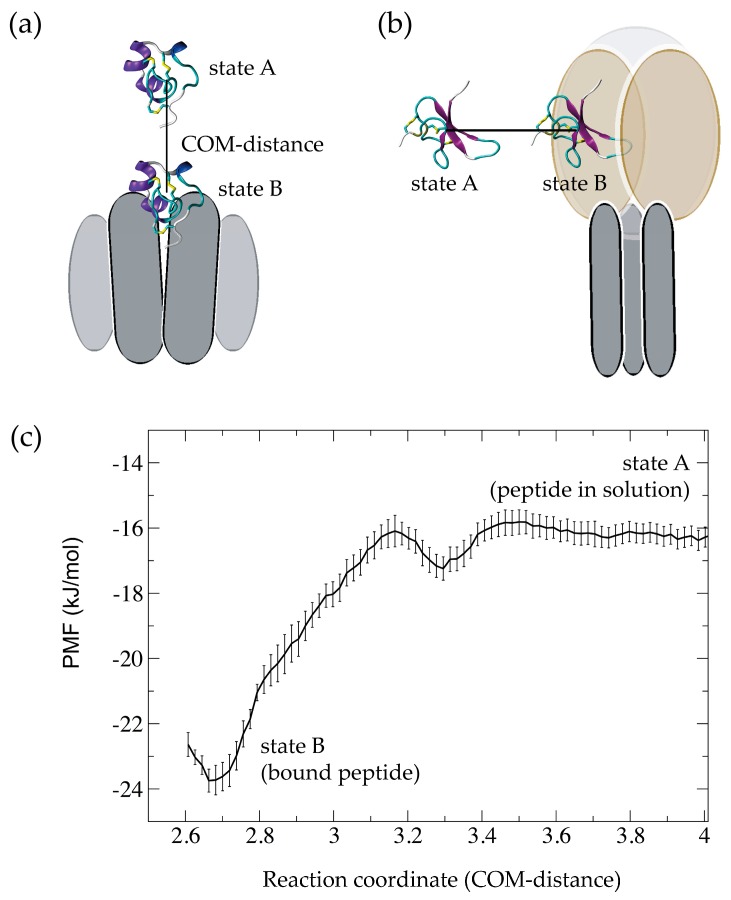
Free energy calculations from umbrella sampling simulations. (**a**,**b**) Schematic of a one-dimensional reaction path that connects the peptide in solution (state A) to the peptide bound to a protein (state B). The reaction coordinate is the centre of mass (COM) distance between the peptide and the protein; (**c**) Example of a potential of mean force (PMF) obtained from umbrella sampling simulations that shows the free energy as a function of the reaction coordinate.

Umbrella sampling simulations are a common method of calculating ΔG_b_ for peptide-channel complexes. In this method, a pathway—referred to as the ‘reaction coordinate’—connects the two states of interest and the free energy difference between the states along that path is determined. For a peptide-channel complex this is often a one-dimensional path connecting the peptide in solution (state A) to the peptide bound to the protein (state B), as illustrated in Figure 3a,b. The reaction coordinate is the distance between the centre of mass (COM) of the peptide and the COM of the channel (COM-distance in Figure 3). To calculate the change in free energy, a series of independent simulations (windows) in which the peptide is restrained to a specific COM-distance are carried out. From these, the free energy as a function of COM-distance—referred to as the ‘potential of mean force’ (PMF)—is calculated (Figure 3c). The PMF can then be used to calculate ΔG_b_ from which the dissociation constant, *K*_d_, can be obtained. A discussion on calculating *K*_d_ from free energy calculations of pore-blocking peptides can be found in a review by Gordon et al. [14]. Note that the binding of peptides is usually reversible and this needs to be reflected in the free energy calculations. Thus, in addition to checking for convergence, the simulations should be checked for reversibility. A semi-quantitative comparison of ΔG_b_ to experimental values or a comparison of ΔG_b_ for different peptides (i.e., ΔΔG_b_) with known differences in activity can give a first indication whether the simulations adequately sample all the required degrees of freedom that contribute to ΔG_b_. For a quantitative comparison between ΔG_b_ and *K*_d_ from simulations and experimental data standard state corrections to calculate the standard binding free energy, ΔG_b_°, might have to be considered [71,74,91,92,93]. 

A number of studies have used this approach to calculate the PMF for the binding or unbinding of a pore blocking peptide to the selectivity filter of a Na_V_ or K_V_ channel [32,34,42,44,47,48,49,59,62,64,65,68,94,95]. In these simulations, the distance between the peptide and the binding pocket along a vector that runs trough the central axis of the channel pore was used as a reaction coordinate (Figure 3a). The level of agreement between experimental *K*_d_ values and the ones calculated from the PMF varies a lot between studies highlighting the challenges involved in the accurate calculation of binding free energies for complex systems. In a recent review of K_V_ blockers, Novoseletsky et al. [16] listed a comparison of computed and experimental binding free energies for 11 peptide-channel systems. Of these, eight are within chemical accuracy of the experimental values (i.e., within 1 kcal/mol or ~4 kJ/mol). Of the remaining three, two are within ~10 kJ/mol and one value shows a difference of more than 60 kJ/mol. Note that a change in ΔG_b_ of ~5.7 kJ/mol is equivalent to a 10-fold change in the equilibrium constant. 

A good agreement between the experimental and calculated ΔG_b_ increases the confidence in the structure of the peptide-channel complex. In these cases, the simulations used to calculate the PMF can provide atomistic level insight into the mechanism of action that is hard to obtain experimentally. For example, PMFs have been used to distinguish between alternative binding modes [49], to understand which residues govern the experimentally observed subtype selectivity of different peptides [59,62,65,96] or to design modified peptides (mutants) that show an increased potency and/or enhanced selectivity for a specific channel [44,62,64,96]. On the other hand, when calculated and experimental values show large discrepancies, it is often not straightforward to determine the cause. In a study of the complex formed by the scorpion-venom peptide ChTx and the potassium channel KcsA, the binding free energy calculated from the PMF was ~30 kJ/mol higher than the experimental data [32]. It was later found that the restraining potential used in the umbrella sampling simulations caused the peptide to be distorted. Correcting for the contribution of this deformation to the overall binding free energy [32] or preventing the deformation using position restraints on the peptide [94] resulted in a significantly better agreement with experimental data. In another study, Khabiri et al. calculated the PMF for the binding of ChTx to *m*K_V_1.3 and the resulting binding free energy was ~38 kJ/mol lower than the experimental data [34]. The authors attributed this discrepancy to the different salt concentrations used in the calculations and the experiments. Many other simulations studies of peptides binding to K_V_ channels in which the salt concentrations were similar to the one used by Khabiri et al. produced PMFs for which the binding free energy was in very good to acceptable agreement with experimental data. A particular problem for Na_V_ channels is that experiments are often carried out on mammalian channels while simulations are carried out using the crystal structure of the bacterial channel (the only Na_V_ crystal structure available) or homology models thereof. Chen et al. [42] calculated the PMF of the α-conotoxin PIIIA binding to the bacterial sodium channel Na_V_Ab and the calculated IC_50_ values were 100–1000 fold lower than the values for the binding of PIIIA to mammalian Na_V_ channels. Based on this data alone, it is not possible to determine whether PIIIA really shows such different affinities for the mammalian and bacterial channel or whether the simulations predicted wrong binding affinities. 

It is also worth noting that in all of the above studies the complex formation did not seem to induce large conformational changes in the channel or the peptide. This reduces the conformational space that needs to be sampled and thus shortens the time required to converge the PMF. In the majority of the simulations described above, convergence was achieved within <10 ns of simulation per window. If the binding involves large conformational changes in the peptide or the channel, much longer simulation times per window would be needed to reach convergence.

Although computationally expensive, umbrella sampling has been the method of choice for calculating the PMF of peptide-channel complexes. A series of recent studies suggested that Jarzynski’s equality in combination with steered MD might provide a more efficient way of calculating the same PMF. This method differs from all other methods presented here in that it is a non-equilibrium work approach. Jarzynski’s equality relates the work done under non-equilibrium conditions to the equilibrium free energy change [97]. In practice, the PMF is calculated from steered MD simulations where a time-dependent but constant force is applied to move the system away from equilibrium [84,85]. The simulation usually starts from the bound peptide and the peptide is slowly steered away from the binding site towards the bulk solvent along a one-dimensional reaction coordinate (again implying the binding/unbinding is reversible). Nonetheless, to obtain an accurate and converged PMF, the system cannot deviate too much from equilibrium. The force applied to the peptide should not be much higher than the relaxation time of the surrounding environment that has to adapt to the peptide being moved away [70]. While the method has been successfully applied to small systems there has not been many studies on large and complex systems [70,84]. Baştuğ et al. [76] compared the PMF for the peptide CnErg1 binding to (a homology model) of the hERG K^+^ channel calculated using umbrella sampling and Jarzynski’s equality. The binding affinity obtained from umbrella sampling simulations is ~35 kJ/mol. The affinity calculated from experimental binding constant is ~47 kJ/mol. In contrast, the binding affinity from the PMF obtain using Jarzynski’s equality is ~250 kJ/mol. The authors concluded that in this case Jarzynski’s method suffers from relaxation problems and that the issues could be overcome by using a slower steering velocity or have a much larger number of samples (windows). Both of these solutions would however increase the simulation time well beyond that of umbrella sampling simulations, thus negating the original motivation for the method. More studies are required to examine whether Jarzynski’s equality is a viable alternative to umbrella sampling for calculating the PMF of peptide-channel complexes.

#### 2.3.2. Predicting the Effect of Mutations on the Binding Affinity 

Site-directed mutagenesis is routinely employed to study the mechanism of action and improve the selectivity or potency of a peptide for a given ion channel. While it is possible to calculate the effect of a mutation on the binding free energy by comparing the PMF of the wild-type and mutant peptide [44,64,65], this is resource-intensive. Free energy perturbation (FEP) can provide a computationally cheaper alternative. This method uses an ‘alchemical’ pathway rather than a reaction coordinate to calculate the difference in free energy between two states. For this, the system is slowly ‘transformed’ from state A (the initial or reference state) into state B (the final or end state). To estimate the effect of mutation on the binding free energy, the modified residue is slowly ‘transformed’ from its wild-type state to its mutated state. For example, a Phe-to-Ala mutation involves the transformation of the Phe side chain into a methyl group. This approach is however restricted to cases where the mutation causes only local changes in peptide-channel interactions and does not change the structural fold of the peptide or its orientation in the binding site. For example, Mahdavi et al. [47] first used umbrella sampling simulations to calculate the PMF for the binding of the peptide *κ*-conotoxin PVIIA to the *Shaker* K^+^ channel. In a second step, the authors aimed to use FEP calculations to predict the change in binding free energy (ΔΔG_b_) for the peptide mutants F9A and F23A. Analysis of the PMF simulations revealed that the F9A mutation changes the structure of peptide, which made the FEP method unfeasible. Nevertheless, the ΔΔG_b_ for the F23A mutant was calculated to be ~9.8 kJ/mol, in good agreement with the experimental value of ~7.8 kJ/mol. Similarly, another simulation study showed that the mutation R29A in the peptide ShK, a potent inhibitor of Kv1.1, changes the peptide’s binding mode, which prevented the use of FEP to calculate ΔΔG_b_ [98]. In fact, mutations of charged residues pose a particular challenge for FEP calculations. To address this issue, Rashid et al. [98] reported a multi-step approach in which the Lennard-Jones interactions are decoupled from the Coulomb interactions and the mutation is simultaneously performed on the residue in the bound and unbound state. The approach was used to calculate the ΔΔG_b_ for the R18A mutation in the peptide-channel complex HsTx1-K_V_1.1 and the data showed good agreement with experiments. 

Despite the reduced cost of FEP, the method is still too expensive to compare the effect of a large number of mutations. For this, endpoint methods with continuum models such as MM/PBSA or MM/GBSA are more feasible. As the name implies, these methods ignore the intermediate states and calculate the difference in free energy simply by comparing the free energies of states A (the channel in complex with the wild-type peptide) and B (the channel in complex with the mutant peptide) [86,87]. Combined with implicit solvation models, this significantly reduces the computational cost for calculating ΔΔG_b_. To still include some conformational sampling, the energy for the peptide-channel complexes are usually averaged over a set of structures obtained from MD simulations. Like FEP, this method is only suitable if the mutation does not significantly alter the binding mode. A number of studies have shown that the method is very sensitive to the parameters used (e.g., the dielectric constant of the solvation model). Also, the accuracy can be very system-dependent (see [86,87,88] and references therein). This is particularly true for large and complex systems that involve binding partners with conformational flexibility and/or charged species; both of which apply to peptide-channel complexes. Studies on different complexes showed that the accuracy of these methods ranges from 4 kJ/mol to 12 kJ/mol. In some cases, this is sufficient to distinguish between alternative binding modes [35,51,53,99,100] or to explain experimentally observed subtype selectivity [52]. Eriksson et al. [67] used the MM/PBSA approach to calculate ΔΔG_b_ for 18 mutants of the complex formed by AgTx2 and the *Shaker* K^+^ channel with the aim to distinguish four alternative binding modes predicted from restrained MD simulations. While the calculated ΔΔG_b_ values showed reasonable agreement with experimental values from double mutant cycles, the accuracy was not sufficient to conclusively distinguish between different binding modes. Instead the authors calculated the coupling constant Ω, the raw data obtained from double mutant cycles—based on these results—were able to rule out two of the four binding modes. 

## 3. Simulations of Peptide-Membrane Interactions 

For peptides binding to solvent accessible domains of an ion channel, it is usually sufficient to characterise the peptide-channel interactions to fully understand the peptide’s mechanism of action. While membrane partitioning is not a prerequisite to act as a gating modifier [101,102,103,104] for some peptides, it is part of the mechanism by which they inhibit voltage-gated ion channels [11,12,105]. Simulation studies of gating modifiers have mainly focused on investigating the depth of penetration or the orientation of the peptide on/in the membrane [11,102,106,107,108,109], identifying the residues that govern membrane binding [11,102], or calculating the change in free energy associated with membrane binding [110,111,112]. 

Accurately predicting the interaction of a peptide with a membrane is even more difficult than for peptide-channel complexes. The membrane surface does not provide a distinct ‘lipid-binding site’. Instead, the peptide can interact with the membrane in multiple ways depending on the conformation of the peptide and the local shape and composition of the membrane. In addition, the ‘interaction sites’ on the membrane are not spatially separate or unique. In principle, the peptide can continue to bind to the membrane until the surface is covered in peptides. This significantly increases the conformational spaces to be sampled. In addition, peptides show different binding affinities depending on the lipid composition of the membrane [11,102,103,105,113]. 

A number of recent studies used MD simulations to determine the position of the peptide on the membrane surface and identify the residues that govern membrane binding. Data was in good qualitative agreement with structural data from experiments [11,18,106,108]. The accurate and reliable prediction of binding affinity from free energy calculations remains very challenging as demonstrated by a number of recent studies. Wee et al. [111,112] aimed to calculate the binding free energy of the gating modifier VsTx1 to neutral and negatively charged phospholipid bilayers. One-dimensional PMFs along a reaction coordinate perpendicular to the membrane were calculated using umbrella sampling simulations. In one study, coarse-grained simulations were used to increase the accessible simulation times. The binding constants calculated from the PMF were orders of magnitudes larger than experimentally observed data. In a subsequent study, PMFs for the same systems were re-estimated using three different representations; a model in which the explicitly modelled peptide was combined with an implicit membrane-solvent model, a coarse-grained and an all-atom model with explicit solvent. The PMFs obtained from the different methods showed a similar overall shape and predicted the peptide to be positioned at the water-lipid interface, which is in agreement with experimental data. The change in binding free energy as well as the energy barriers predicted from the different methods showed significant differences and none of them agreed with experimental values. In another study, Chen et al. [110] used umbrella sampling simulations to calculate the PMF for the binding of the peptides GsMtx4 and HpTx2 to neutral phospholipid bilayers. In agreement with experimental data [103], the relative binding affinity was predicted to be much lower for HpTx2 (~30 kJ/mol) than for GMTx4 (~60 kJ/mol). For both peptides, the binding affinities from the simulations were more than 10 kJ/mol larger than experimental values. 

## 4. Summary

Disulfide-rich peptides isolated from the venom of arthropods and marine animals are potent and selective inhibitors of ion channels making them valuable lead molecules for the development of drugs and pharmacological tools. Consequently, much effort goes into understanding their mechanism of action. Simulations have contributed to this by enabling the study of the interactions between the peptide and an ion channel or membrane at the molecular level. A number of studies have used simulation approaches to predict the structure of peptide-channel complexes, calculate the free energy of binding for wild-type and mutant peptides, and provide insights into the membrane-binding activities of gating modifier peptides. With careful validation, structural models from simulations can provide mechanistic insight not available from experiment. This information can then be used to rationalise the experimentally observed potency and selectivity of the peptide and design modified peptides for specific applications. 

The review of these simulation studies has highlighted the challenges that come with modelling large and complex biomolecular systems. Simulations of peptide-channel and peptide-membrane systems are still very resource intensive, require in-depth knowledge of a range of simulation techniques, and heavily rely on the availability of experimental data to guide the simulation and/or for validation of predicted binding modes. Current state-of-the art methods prevent the screening of large numbers of peptides and are more suitable for late-stage lead optimisation. The continuing increase in computing power and the on-going efforts in improving docking methods and biomolecular force fields means that, in the future, simulations might be routinely used for the rational design of venom-based peptides. 

Due to the lack of high-resolution structures of ion channels simulation studies have mostly been limited to complexes formed by disulfide-rich venom peptides and a small number of voltage-gated Na^+^ and K^+^ channels and acid sensing ion channels. The rapid advances in electron microscopy are likely to increase the number of high-resolution structures of membrane proteins and peptide-channel complexes. This will certainly result in an increased use of simulation methods to study these systems. It has been estimated that less than 0.01% spider venom peptides have been described and even less have been fully characterised [114]. Simulations and other computational approaches will undoubtedly play an important role in harnessing the therapeutic potential of these remarkable peptides.
